# Effectiveness of Stabilization of Preterm Infants With Intact Umbilical Cord Using a Purpose-Built Resuscitation Table—Study Protocol for a Randomized Controlled Trial

**DOI:** 10.3389/fped.2019.00134

**Published:** 2019-04-12

**Authors:** Ronny Knol, Emma Brouwer, Frans J. C. M. Klumper, Thomas van den Akker, Philip DeKoninck, G. J. Hutten, Enrico Lopriore, Anton H. van Kaam, Graeme R. Polglase, Irwin K. M. Reiss, Stuart B. Hooper, Arjan B. te Pas

**Affiliations:** ^1^Division of Neonatology, Department of Pediatrics, Erasmus MC University Medical Center, Rotterdam, Netherlands; ^2^Division of Neonatology, Department of Pediatrics, Leiden University Medical Center, Leiden, Netherlands; ^3^Department of Obstetrics, Leiden University Medical Center, Leiden, Netherlands; ^4^Department of Obstetrics and Gynecology, Erasmus MC University Medical Center, Rotterdam, Netherlands; ^5^The Ritchie Centre, Hudson Institute of Medical Research, Monash University, Clayton, VIC, Australia; ^6^Department of Neonatology, Emma Children's Hospital, Amsterdam UMC, University of Amsterdam, Amsterdam, Netherlands

**Keywords:** preterm infants, resuscitation, umbilical cord clamping, newborn transition, physiological-based cord clamping, randomized controlled trial, study protocol

## Abstract

**Background:** Most preterm infants fail to aerate their immature lungs at birth and need respiratory support for cardiopulmonary stabilization. Cord clamping before lung aeration compromises cardiovascular function. Delaying cord clamping until the lung has aerated may be beneficial for preterm infants by optimizing hemodynamic transition and placental transfusion. A new purpose-built resuscitation table (the Concord) has been designed making it possible to keep the cord intact after preterm birth until the lung is aerated and the infant is respiratory stable and breathing [Physiological-Based Cord Clamping (PBCC)]. The aim of this study is to test the hypothesis whether stabilizing preterm infants by PBCC is at least as effective as the standard approach using time-based Delayed Cord Clamping (DCC).

**Study design:** This is a randomized controlled non-inferiority study including 64 preterm infants born at <32 weeks of gestation. Infants will be randomized to either the PBCC approach or standard DCC. In case of PBCC, infants will be stabilized with an intact umbilical cord and the cord will only be clamped when the infant is considered respiratory stable, defined as the establishment of regular spontaneous breathing, a heart rate ≥100 bpm and oxygen saturation above 90% while using inspired fraction of oxygen (FiO2) < 0.40. The Concord will be used, which allows giving respiratory support with an intact umbilical cord. In the DCC group infants are clamped first before they are transferred to the standard resuscitation table for further treatment and stabilization. Cord clamping is time-based and delayed at 30–60 s. The primary outcome will be the time to respiratory stability of the infant, starting from birth. Secondary outcomes will include details of stabilization, important clinical outcomes of prematurity and maternal safety outcomes.

**Discussion:** We expect that PBCC using the Concord may reduce major morbidities and mortality in preterm infants. The current study protocol will assess the effectivity of stabilization. Once effectivity of stabilization is confirmed, we will start a large multicenter randomized clinical trial to investigate whether PBCC reduces mortality and morbidity in preterm infants compared to the standard approach.

**Trial registration:** Netherlands Trial Registry NTR7194, registered on April 20th, 2018.

## Introduction

Preterm infants are most vulnerable immediately after birth. Management in the first minutes of life can have a major impact on important short and long-term morbidities associated with prematurity (1, 2). During the transition to extra-uterine life, lung aeration at birth is pivotal for the major physiological changes in respiratory and cardiovascular function that are required for survival after birth (3, 4). Most preterm infants fail to aerate their immature lungs and require respiratory support at birth (5). This is commonly conducted after umbilical cord clamping, which in experimental studies has shown to compromise cardiovascular function (6).

Preterm infants could benefit from a placental transfusion (blood from the placenta to the infant) when cord clamping is delayed. A recent meta-analysis comparing delayed cord clamping (DCC) with immediate cord clamping (ICC) in preterm infants indeed showed an increased hematocrit, less blood transfusions, a decrease in neonatal mortality, and a trend toward less intraventricular hemorrhages (IVH) (7). However, in most studies DCC was performed using a fixed time-point of 30–60 s while placental transfusion is only complete after 3 min (8). In addition, preterm infants needing immediate interventions for stabilization or resuscitation were clamped immediately and thus excluded, while these infants have the highest risk of complications.

While the rationale of most cord clamping studies was based on increased placental transfusion, we recently demonstrated in preterm lambs that delaying cord clamping until ventilation onset prevents a significant reduction in cardiac output (6). This also prevented large fluctuations in systemic and cerebral blood pressures and flows, and concomitant bradycardia and hypoxia after ICC (9). This finding may explain the bradycardia and hypoxia that is often observed in preterm infants after ICC (10–12). Avoiding these adverse effects may decrease the risk of mortality and (cerebral) morbidity (13). The adverse effects could possibly be avoided when preterm infants are first stabilized with the cord intact and only clamped when the infant is respiratory stable. We called this physiological-based cord clamping (PBCC) as the moment of cord clamping is based on the clinical condition of the infant (14).

Some studies in preterm infants have been performed where respiratory support was provided before the cord was clamped. These protocols all used a time-based approach, with cord clamping at 60 s, 90 s, and 2 min after birth, respectively (15–17). PBCC is based on the clinical condition of the infant, but no clear criteria are available to define when an infant is respiratory stable. We will use the definition that the infant has established regular spontaneous breathing with oxygen saturation (SpO2) above 90%, heart rate > 100, and oxygen need < 40%. This is based on previous observations of stabilization of preterm infants showing that most infants were breathing with CPAP within 5 min after birth (18). When performing PBCC the primary aim is lung aeration and sufficient increase in pulmonary blood flow before the cord is clamped and by using our definition we expect to be able to achieve these aims.

**Figure 1:**
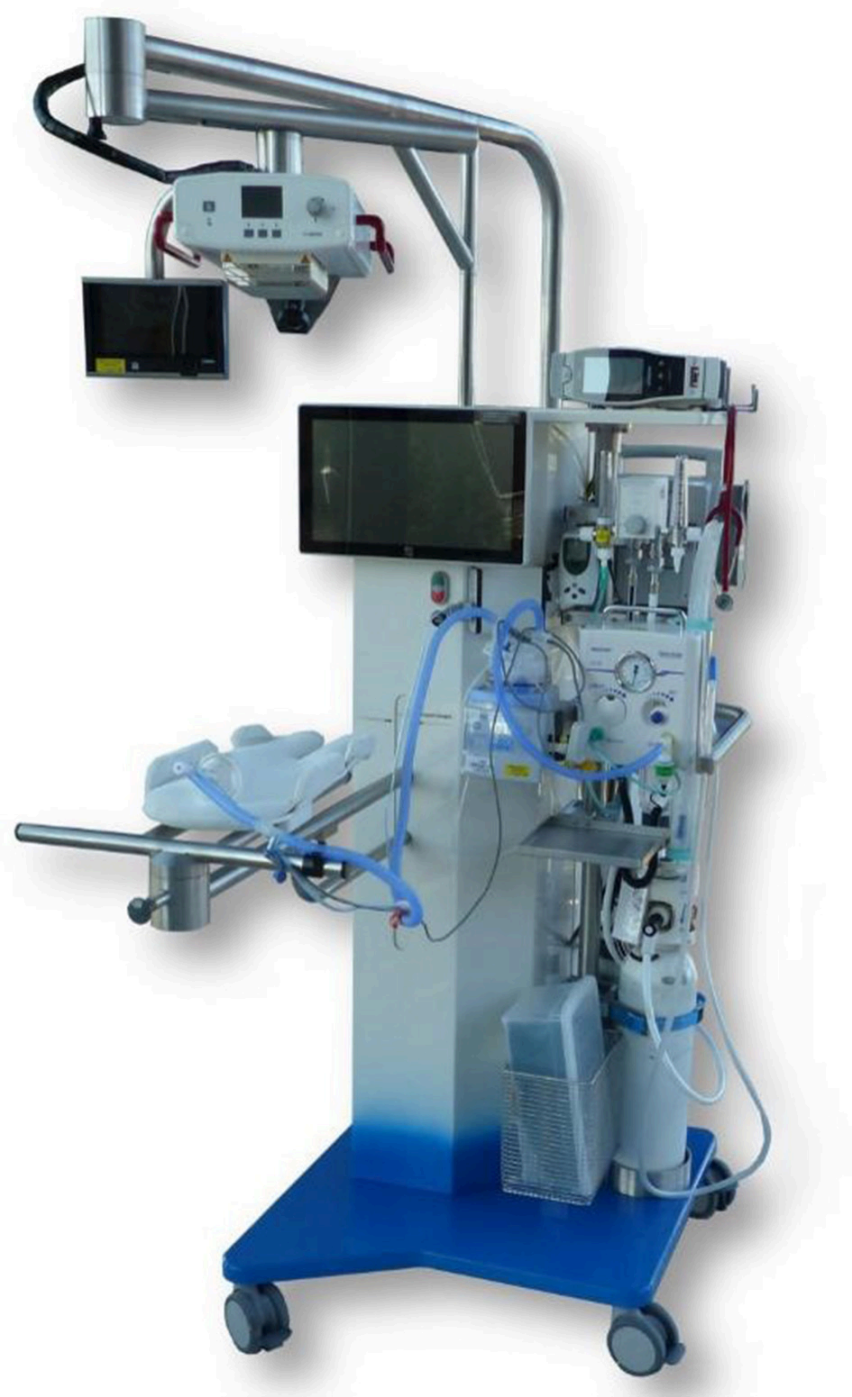
The Concord, a mobile resuscitation table specially designed to provide full standard care in stabilization of preterm infants at birth while the cord remains intact.

Leiden University Medical Center (LUMC) developed a new purpose-built resuscitation table, the Concord (Figure 1). This mobile resuscitation table is designed to provide full standard care in stabilization of preterm infants at birth while the cord remains intact. All equipment that is needed for stabilization and resuscitation is incorporated in the table (www.concordneonatal.com). We recently completed a feasibility study in preterm infants demonstrating that PBCC using the Concord was feasible and safe (19). We observed less bradycardia and hypoxia at birth, supporting the more stable hemodynamic transition observed in animal studies. In addition, the average cord clamping time was more than 4 min, which may allow preterm infants to benefit from a more optimal placental transfusion. This protocol is designed to assess the effectivity of the procedure and to test whether stabilization of preterm infants according to PBCC using the Concord is at least as effective as stabilization according to the standard approach using the standard table.

### Aim of the Study

To investigate whether stabilization of preterm infants according to PBCC using the Concord is at least as effective as stabilization according to the current routine approach using a standard resuscitation table.

## Study Design and Population

### Study Design

**Figure 2:**
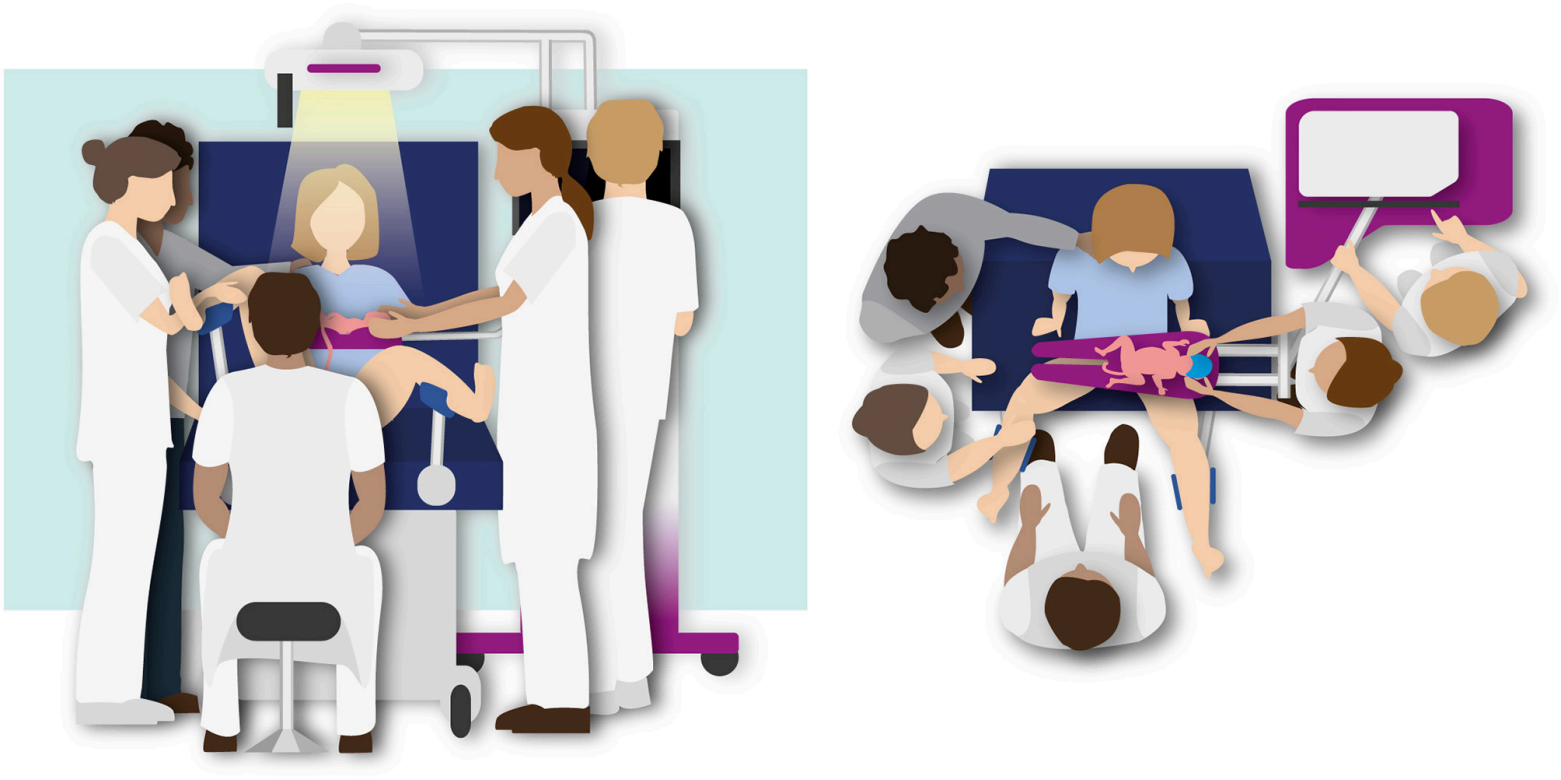
Illustration of the Physiological Based Cord Clamping approach using the Concord. Stabilization of the infant is performed while the cord is intact and the cord will be clamped after the infant is respiratory stable.

**Figure 3:**
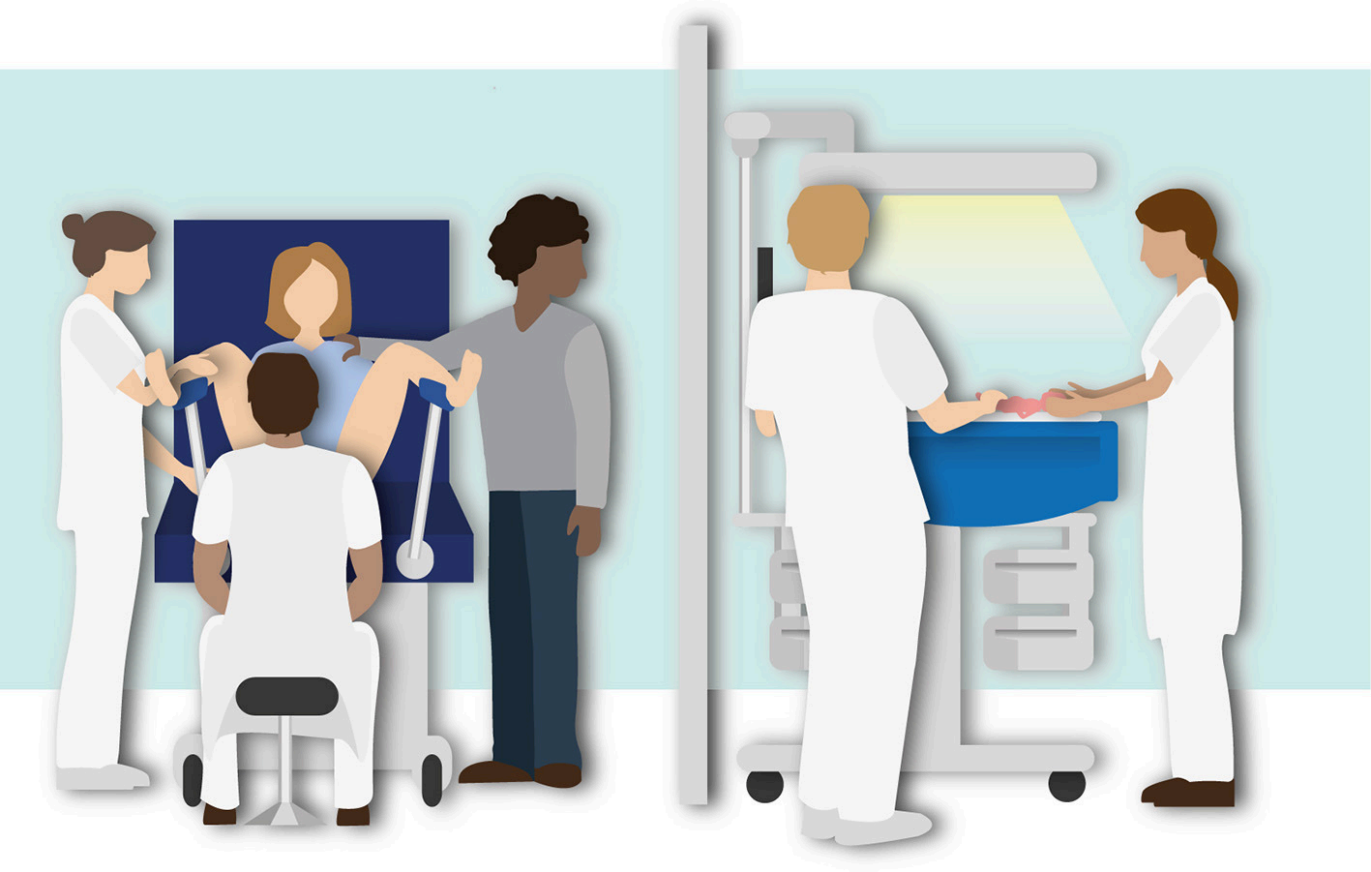
Illustration of the routine Delayed Cord Clamping approach using the standard resuscitation table. Stabilization of the infant is performed after delayed cord clamping (30–60 s).

Randomized controlled non-inferiority study in three centers (Leiden University Medical Center, Erasmus MC University Medical Center and Amsterdam University Medical Centers). The study will be performed in the delivery room or operating room in case of a cesarean section. Infants will be randomized to either stabilization according to PBCC using the Concord (Figure 2), or according to the standard approach using the standard resuscitation table (Figure 3). The study is approved by the LUMC Institutional Review Board (IRB, P18.025) and is registered in the Netherlands Trial Register (NTR7194).

### Study Population

Infants born prior to 32 weeks of gestational age in one of the three centers may be included in the study. Vaginally and cesarean delivered infants may be included.

Exclusion criteria are significant congenital malformations influencing cardiopulmonary transition, signs of placental abruption or placenta praevia and signs of severe fetal distress necessitating an emergency cesarean section.

Baseline characteristics will be recorded, including gestational age, birth weight, sex, and single or twin deliveries.

### Primary Outcome

The primary outcome will be the time needed to stabilize the infant, starting from birth. Stable is defined as the establishment of regular spontaneous breathing, a heart rate ≥ 100 bpm and oxygen saturation above 90% while using FiO2 < 0.40.

### Secondary Outcomes

Multiple secondary outcomes will be collected, concerning transition of the infant as well as short-term neonatal and maternal outcomes and the procedure itself.

#### Outcomes Relating to the Transition of the Infant

- The time point after birth when respiratory support was started and oximeter signals could be interpreted
- The occurrence the PBCC approach could not be performed and reason why
- Failure of reaching respiratory stability within 10 min from birth
- Duration of mask ventilation given
- Average pressures given during mask ventilation
- Average pressure given during mask Continuous Positive Airway Pressure (CPAP)
- Oxygen saturation and heart rate in the first 10 min from birth
- Time point of cord clamping
- Problems occurring with the cord before cord clamping
- Occurrence of the need for cord clamping before stabilization
- The need for intubation in the delivery room
- The rate of hypothermia at admission in the NICU
- Apgar scores at 1, 5, and 10 min
- Echocardiography measurement: ductal flow ratio at 1 h of age

#### Short-Term Maternal Outcomes

- Perinatal blood loss
- The rate of postpartum hemorrhage
- The rate of surgical site wound infection after cesarean section

#### Short-Term Neonatal Outcomes

- Mortality at 28 days postnatal age and at NICU discharge
- Hemoglobin and hematocrit levels within 24 h
- Need for intubation and mechanical ventilation in the NICU
- Surfactant treatment
- Frequency and duration of phototherapy
- Number of erythrocyte transfusions
- Necrotizing enterocolitis
- Intraventricular hemorrhage
- Bronchopulmonary dysplasia
- Retinopathy of prematurity

The parents and the health care providers will be asked to fill in a short questionnaire concerning the birth and stabilization. The questionnaire will also contain questions whether they were satisfied and felt comfortable with the approach.

### Sample Size Calculation

There is no data available concerning the effectiveness of stabilization during PBCC and comparing PBCC with standard approaches. Few studies have performed initial respiratory support before cord clamping, but all of them used predefined timing of cord clamping, varying from 30 to 120 s. Data from our feasibility study showed a median time to obtain respiratory stability of 4:23 min [IQR 3:00–5:11] when PBCC is performed.

Using the routine approach the mean time needed to stabilize an infant, starting from birth, is 5 ± 2 min (unpublished data, Leiden database). If there is truly no difference for the time needed to stabilize an infant between the standard approach and PBCC, then 64 infants (32 in each treatment arm) are required to have 80% power to assess whether the lower limit of a one-sided 97.5% confidence interval (or equivalently a 95% two-sided confidence interval) around the difference in the mean-time to stabilization (mean time in the standard group minus mean time in the experimental treatment group) will be above the non-inferiority limit of −75 s. We have chosen the non-inferiority level of 75 s, because this difference is still acceptable according to the current clinical guidelines. We will take in consideration that 7% of infants (unpublished data, Leiden database) will not reach our primary endpoint set for stabilization, because they need continuous PPV, with or without intubation. These infants will be included in all intended analyses.

## Treatment of Subjects and Investigational Equipment

Prior to the start of the study all health care providers involved in delivery room care will be trained in using the Concord for PBCC. A standard operating procedure has been developed to optimize close collaboration between neonatologist and obstetrician. All neonatal caregivers involved are trained and accredited for neonatal resuscitation. Centers will adhere to local resuscitation guidelines for stabilization.

### Intervention

Preterm infants randomized to the intervention group will be stabilized according to PBCC. With the exception that the infant is stabilized close to the mother and the cord is clamped at a later stage, the infant will receive standard postnatal respiratory management and heat loss prevention.

#### Physiological-Based Cord Clamping

The Concord will be placed next to the bed of the mother and all equipment will be checked before the second stage of labor has started or prior to cesarean section. Stabilization will be started as soon as the infant is placed on the platform and according to the local resuscitation guidelines.

When an infant is randomized to PBCC, the standard resuscitation table will also be set and prepared for use. In any case if the attending neonatologist and/or obstetrician decides that PBCC should not be performed or interrupted, the infant can be taken to the standard resuscitation table for (further) stabilization.

##### Timing of cord clamping, performing PBCC

Stabilization of the infant is performed while the cord is intact and the cord will be clamped after the infant is respiratory stable, defined as the establishment of regular spontaneous breathing, a heart rate >100 bpm and oxygen saturation above 90% while using supplemental oxygen < 40%.

##### Summary of standard operating procedure PBCC

- The Concord is placed next to the bed of the mother, preferably at the left side.
- After the infant is born, the obstetrician holds the baby.
- The platform of the table is placed as close as possible to the mother's pelvis to make sure that stretching of the umbilical cord will not occur.
- The infant is placed on the platform and receives stabilization and heat loss prevention according to standard guidelines. The nurse places the oximeter sensor on the right wrist of the baby.
- In case of vaginal delivery, the mother is able to touch and stimulate the infant during stabilization. The nurse communicates with the parents to explain the progress and procedures.
- The cord is clamped as soon as the infant is respiratory stable, as defined before.
- To ensure optimal placental transfusion the minimum clamping time is 3 min.
- If the infant does not reach the criteria for being stable, the maximum clamping time is 10 min.
- After clamping, the platform will be withdrawn and placed next to the bed of the mother.
- Uterotonic drugs are administered immediately after cord clamping.
- The infant will be prepared and transferred to the transport incubator.

In case of twins and vaginal delivery, the same definition of the moment of cord clamping will be used. Clamping in the first infant will be performed sooner if the second infant of the twins is about to be born. The first infant will then be transferred to the standard resuscitation table, so that the second twin can be stabilized on the Concord.

PBCC can be aborted at any time during the procedure. The cord will be clamped and the infant is transferred to the standard resuscitation table:

- When an emergency occurs with mother or second twin and more working space is necessary for the obstetrician.
- When full resuscitation is needed and the parents have expressed this should not be performed close in their view.
- When maternal blood loss is excessive and the obstetrician decides to clamp the cord and to administer uterotonic drugs immediately.

These infants remain included in the study, as this is an outcome parameter.

### Standard Treatment

Preterm infants are clamped first and then transferred to the standard resuscitation table for further treatment and intervention needed for cardiopulmonary stabilization, according to the local resuscitation guidelines. Clamping is time based and performed immediately or delayed at 30–60 s, depending on the clinical condition of the infant. Uterotonic drugs are administered immediately after cord clamping.

### Investigational Equipment

The Concord is specially designed to provide full standard care in stabilization of preterm infants at birth while the cord remains intact. Via an extendable arm, the support platform can be placed very close to the mother so that a preterm infant, independent of the length of the umbilical cord, can receive PBCC. A patented slit is incorporated in the working plate in which the umbilical cord can be placed, thereby preventing stretch of the umbilical cord. All equipment that is needed for stabilization and resuscitation is incorporated in the table.

For standard care a routine resuscitation table is used. This table is normally situated in the resuscitation room next to the delivery room.

Both tables are provided with standard equipment for stabilization and resuscitation:

- T-piece ventilator
- Radiant heater
- Suctioning device
- Oxygen blender
- Heater and humidifier
- Heated resuscitation circuit
- Pulse oximeter

Both tables have a respiratory function monitor built in. The monitor depicts respiratory function, the applied respiratory support, oxygen saturation, heart rate, FiO2, and a video connected to an MRT-A RFM (Applied Biosignals, Weener, Germany).

## Procedures

### Recruitment and Consent

Parental written informed consent will be obtained before birth. Parents of an eligible infant will be informed by the attending obstetrician or neonatologist and asked for their consent after they have read the information letter.

### Randomization, Blinding and Treatment Allocation

Allocation will be stratified by gestational age (24–27+6 and 28–31+6 weeks) and by treatment center using variable block (4–8) sizes. Concealment of allocation will be ensured by using the randomization process of Castor EDC, an electronic data capture system. The neonatal consultant in charge of the procedure will randomize using Castor EDC. Blinding of the study is not possible.

In case of twin vaginal delivery, both infants will be randomized to the same group. In case of a cesarean section for twins, it is technically not possible to perform PBCC for both infants. After consent, both infants will be included; the first infant will receive standard treatment and the second infant will be randomized to either PBCC or standard treatment.

### Withdrawal of Subjects

Parents and caregivers can withdraw consent at any time and for any reason if they wish to do so without any consequences. All withdrawn infants will receive regular treatment as usual.

### (Serious) Adverse Events

Adverse events are defined as any undesirable experience related to PBCC occurring to a subject during the study. All adverse events observed by the researcher or the staff will be recorded and reported to the IRB.

This study population has a high risk of serious complications (so-called “context-specific serious adverse events (SAEs)”), which are inherent to their vulnerable condition and unrelated to the intervention which is under evaluation in this trial. These complications are included in the secondary outcomes of this study and are recorded in the Case Report Form. Yearly, an overview of the context-specific SAEs for each treatment arm will be presented to the IRB.

Stabilization of preterm infants with the resuscitation table as close as possible to the mother has been performed before and is considered safe. The present study uses a similar approach, using the following modifications: (1) stretching of the umbilical cord is prevented, (2) full standard care for stabilization can be provided, and (3) extended infant monitoring is included. We do not expect that PBCC poses extra risks to standard stabilization of the preterm infant. Nevertheless, we have included several “safety parameters” as secondary outcomes. The two most important safety parameters are maternal blood loss and admission temperature of the infant at the NICU.

This is a small trial where only 32 infants will be included in each group and a Data Safety Monitoring Committee will not be installed. All SAEs mentioned above will be recorded in the Case Report Form. All SAEs will be reported to the IRB. Any unforeseen SAEs that was life threatening or resulted in death and was directly related to the PBCC approach will be reported without undue delay after obtaining knowledge of the event.

### Statistical Analysis

Normally distributed data will be presented as means ± standard deviations, not-normally distributed data as medians and interquartile ranges. Categorical data will be analyzed using the Chi-square test. Continuous data will be analyzed using the Student's *t*-test or Mann-Whitney test as appropriate. Intention-to-treat analysis and per-protocol analysis will be performed. The effect of PBCC on the primary outcome will be assessed by multi-variable logistic regression analysis including possible confounders.

### Data Handling and Study Monitoring

Data will be coded, using a unique code for each participant. No personal identifiers are used in the code. The key to the codes will be safeguarded by the researchers. Video's will be recorded of the procedures in which the infant is fully visible, but no other data will be recorded which can lead to identification of the infant or the supervising doctors and nurses in charge of the procedure. Data will be kept on an institutional research network location of the principal investigator secured with a password. Data will be stored for 15 years according to legislation. Independent study monitoring will take place, according to institutional prescriptions.

### Ethical Considerations

The study will be conducted according to the principles of the Declaration of Helsinki and in accordance with the Medical Research Involving Human Subjects Act (WMO).

In this study most preterm infants need stabilization at birth and might benefit from DCC. Delayed cord clamping has been incorporated in international guidelines. So far, stabilization with the cord intact has been considered a safe approach. We do not expect that there is an added risk as the Concord is fully equipped for stabilization and resuscitation.

### Dissemination of Results

The results of the trial will be published in a peer-reviewed journal and will be presented at national and international conferences. The trial began recruiting in June 2018. It is expected that recruitment for the study will be completed late 2018.

## Discussion

Most preterm infants fail to aerate their immature lungs and need respiratory support for survival. The umbilical cord needs to be clamped first before intervention can be started for cardiopulmonary stabilization. This approach compromises cardiovascular function and placental transfusion in experimental setting (6, 9, 20). Currently, cord clamping in preterm infants is time-based and performed immediately or delayed for 30–60 s, depending on the clinical condition of the infant (5, 21).

Studies have been performed where ventilation was given to very preterm infants while the cord remained intact. However, in these studies cord clamping was time-based and not based on the cardiorespiratory condition of the infant. Katheria et al. compared two different approaches of DCC (15). In this study 150 preterm infants <32 weeks of gestation were randomized to ventilation or stimulation before the cord was clamped at 60 s. Early clamping was performed when the infant was assessed as too unstable. It is unclear whether the infants were stable at the time of cord clamping as they were not monitored during the procedure except for end tidal CO_2_ change. No differences in hematocrit (primary outcome) or neonatal morbidities (secondary outcome) were observed.

Duley et al. performed a continued pilot study in 261 infants <32 weeks of gestation comparing DCC (2 min) with ICC (20 s) (17). No formal sample size was calculated. Ventilation was given in the DCC group, but again cord clamping was based on time and not on the stability of the infant. In a large proportion (40%) of infants randomized to the DCC group the cord was clamped earlier than 2 min. The most common reason for earlier clamping was a short umbilical cord. The authors reported a non-significant decrease in neonatal death and IVH (primary outcomes) in the DCC group and no differences in other morbidities.

Winter et al. performed a pilot study to test the feasibility of ventilation before cord clamping (16). There was no comparison group and clamping was done at a fixed time point (90 s). The authors concluded that ventilation before cord clamping was challenging, but feasible and that randomized clinical trials are warranted to determine clinical benefit.

We recognized that the length of the umbilical cord has been a limiting factor so far in a large proportion of infants. Also, providing standard respiratory care and thermoregulation while the infant remains on the cord proved challenging. To overcome these limitations we designed the Concord. For the design the following preconditions were used: (1) complete standard care in stabilization of the infant can be provided, (2) the vital parameters of the infant can be monitored, (3) possibility to stabilize infants even when the cord is very short, without stretching or kinking of the cord, (4) no interference of the working field of the neonatal care provider with the obstetrical care of the mother. The prototype of the Concord is designed at the Leiden University Medical Center (the Netherlands) and is not commercially available. However, a start-up company (Concord Neonatal), has been launched in 2017 for the commercialization of the Concord.

We expect that PBCC using the Concord may reduce major morbidities and mortality in preterm infants. Feasibility and safety were assessed in an earlier study, for which the results were published recently (19). The current study protocol will assess effectivity of stabilization. We expect to show that the time to stabilization performing PBCC using the Concord will not be longer than in the control group. Once this hypothesis is confirmed, we will start a large multicenter randomized clinical trial to investigate whether PBCC reduces mortality and morbidity in preterm infants compared to the standard approach. Hence, this study protocol is the second step in the process of collecting clinical evidence for this promising improvement in stabilization of preterm infants at birth.

## Author Contributions

RK, EB, and AP wrote the study protocol and all authors participated in reviewing the protocol. RK, EB, FK, TA, PD, EL, GP, SH, and AP participated in building and designing the study. RK, EB, TA, PD, and GH coordinate the study, train the clinicians, and collect and analyze the data. RK wrote the first draft and submitted the article. All authors participated in reviewing and editing the manuscript. All authors have read and approved the final version of this manuscript.

### Conflict of Interest Statement

The authors declare that the research was conducted in the absence of any commercial or financial relationships that could be construed as a potential conflict of interest.

## Abbreviations

CPAP: Continuous Positive Airway Pressure
DCC: Delayed Cord Clamping
GA: Gestational Age
ICC: Immediate Cord Clamping
IRB: Institutional Review Board
IVH: Intraventricular Hemorrhage
NEC: Necrotizing Enterocolitis
NICU: Neonatal Intensive Care Unit
PBCC: Physiological-Based Cord Clamping
PPV: Positive Pressure Ventilation
SAE: Serious Adverse Event
SpO2: Pulse oximetry.

## References

[1] Polglase GR Miller SL Barton SK Baburamani AA Wong FY Aridas JD. Initiation of resuscitation with high tidal volumes causes cerebral hemodynamic disturbance, brain inflammation and injury in preterm lambs. PLoS ONE. (2012) 7:e39535. 10.1371/journal.pone.0039535 22761816 PMC3382197

[2] Polglase GR Miller SL Barton SK Kluckow M Gill AW Hooper SB. Respiratory support for premature neonates in the delivery room: effects on cardiovascular function and the development of brain injury. Pediatr Res. (2014) 75:682–8. 10.1038/pr.2014.40 24614803

[3] Lang JA Pearson JT te Pas AB Wallace MJ Siew ML Kitchen MJ. Ventilation/perfusion mismatch during lung aeration at birth. J Appl Physiol (1985). (2014) 117:535–43. 10.1152/japplphysiol.01358.2013 24994883

[4] Siew ML Wallace MJ Kitchen MJ Lewis RA Fouras A Te Pas AB. Inspiration regulates the rate and temporal pattern of lung liquid clearance and lung aeration at birth. J Appl Physiol (1985). (2009) 106:1888–95. 10.1152/japplphysiol.91526.2008 19342434

[5] Sweet DG Carnielli V Greisen G Hallman M Ozek E Plavka R. European consensus guidelines on the management of respiratory distress syndrome - 2016 update. Neonatology. (2017) 111:107–25. 10.1159/000448985 27649091

[6] Bhatt S Alison BJ Wallace EM Crossley KJ Gill AW Kluckow M. Delaying cord clamping until ventilation onset improves cardiovascular function at birth in preterm lambs. J Physiol. (2013) 591:2113–26. 10.1113/jphysiol.2012.250084 23401615 PMC3634523

[7] Fogarty M Osborn DA Askie L Seidler AL Hunter K Lui K. Delayed vs early umbilical cord clamping for preterm infants: a systematic review and meta-analysis. Am J Obstet Gynecol. (2018) 218:1–18. 10.1016/j.ajog.2017.10.231 29097178

[8] Yao AC Hirvensalo M Lind J. Placental transfusion-rate and uterine contraction. Lancet. (1968) 1:380–3. 10.1016/S0140-6736(68)91352-4 4169972

[9] Polglase GR Dawson JA Kluckow M Gill AW Davis PG Te Pas AB. Ventilation onset prior to umbilical cord clamping (physiological-based cord clamping) improves systemic and cerebral oxygenation in preterm lambs. PLoS ONE. (2015) 10:e0117504. 10.1371/journal.pone.0117504 25689406 PMC4331493

[10] White LN Thio M Owen LS Kamlin CO Sloss S Hooper SB. Achievement of saturation targets in preterm infants <32 weeks' gestational age in the delivery room. Arch Dis Child Fetal Neonatal Ed. (2017) 102:F423–7. 10.1136/archdischild-2015-310311 28302696

[11] Goos TG Rook D van der Eijk AC Kroon AA Pichler G Urlesberger B. Observing the resuscitation of very preterm infants: are we able to follow the oxygen saturation targets? Resuscitation. (2013) 84:1108–13. 10.1016/j.resuscitation.2013.01.025 23376585

[12] Phillipos E Solevag AL Aziz K van OS Pichler G O'Reilly M. Oxygen saturation and heart rate ranges in very preterm infants requiring respiratory support at birth. J Pediatr. (2017) 182:41–6. 10.1016/j.jpeds.2016.11.014 27939259

[13] Oei JL Finer NN Saugstad OD Wright IM Rabi Y Tarnow-Mordi W. Outcomes of oxygen saturation targeting during delivery room stabilisation of preterm infants. Arch Dis Child Fetal Neonatal Ed. (2018) 103:F446–54. 10.1136/archdischild-2016-312366 28988158 PMC6490957

[14] Knol R Brouwer E Vernooij ASN Klumper F DeKoninck P Hooper SB. Clinical aspects of incorporating cord clamping into stabilisation of preterm infants. Arch Dis Child Fetal Neonatal Ed. (2018) 103:F493–7. 10.1136/archdischild-2018-314947 29680790 PMC6109247

[15] Katheria A Poeltler D Durham J Steen J Rich W Arnell K. Neonatal resuscitation with an intact cord: a randomized clinical trial. J Pediatr. (2016) 178:75–80.e3. 10.1016/j.jpeds.2016.07.053 27574999 PMC5527831

[16] Winter J Kattwinkel J Chisholm C Blackman A Wilson S Fairchild K. Ventilation of Preterm Infants during Delayed Cord Clamping (VentFirst): A Pilot Study of Feasibility and Safety. Am J Perinatol. (2017) 34:111–6. 10.1055/s-0036-1584521 27305177

[17] Duley L Dorling J Pushpa-Rajah A Oddie SJ Yoxall CW Schoonakker B. Randomised trial of cord clamping and initial stabilisation at very preterm birth. Arch Dis Child Fetal Neonatal Ed. (2018) 103:F6–14. 10.1136/archdischild-2016-312567 28923985 PMC5750367

[18] Huberts TJP Foglia EE Narayen IC van Vonderen JJ Hooper SB Te Pas AB. The breathing effort of very preterm infants at birth. J Pediatr. (2018) 194:54–9. 10.1016/j.jpeds.2017.11.008 29336795

[19] Brouwer E Knol R Vernooij ASN Van den Akker T Vlasman PE Klumper FJCM. Physiological-based cord clamping in preterm infants using a new purpose-built resuscitation table; a feasibility study. Arch Dis Child Fetal Neonatal Ed. (2018). 10.1136/archdischild-2018-315483. [Epub ahead of print]. 30282674 PMC6764254

[20] Blank DA Polglase GR Kluckow M Gill AW Crossley KJ Moxham A. Haemodynamic effects of umbilical cord milking in premature sheep during the neonatal transition. Arch Dis Child Fetal Neonatal Ed. (2018) 103:F539–46. 10.1136/archdischild-2017-314005 29208663 PMC6278653

[21] World Health Organization Guideline: Delayed Umbilical Cord Clamping for Improved Maternal and Infant Health and Nutrition Outcomes. Geneva: World Health Organization (2014). 26269880

